# Encapsulated Peritoneal Sclerosis Masquerading as an Abdominal Catastrophe in Peritoneal Dialysis Therapy

**DOI:** 10.7759/cureus.12934

**Published:** 2021-01-27

**Authors:** Justin Leeoloy, Mayanka Kambojia, Aparna Wagle Shukla, Xuili Liu, Ashutosh Shukla

**Affiliations:** 1 Medicine, University of Florida College of Medicine, Gainesville, USA; 2 Medicine/Nephrology, University of Florida, Gainesville, USA; 3 Neurology, University of Florida, Gainesville, USA; 4 Pathology, University of Florida, Gainesville, USA; 5 Medicine/Nephrology, North Florida/South Georgia (NF/SG) Veteran Healthcare System, Gainesville, USA

**Keywords:** encapsulating peritoneal sclerosis, acute abdomen, peritoneal dialysis (pd)

## Abstract

Encapsulated peritoneal sclerosis (EPS) is a rare but known complication of peritoneal dialysis (PD) therapy in patients with end-stage renal disease (ESRD). It commonly manifests insidiously with recurrent intestinal obstruction and malnutrition, worsening over time. We report an ESRD patient on PD therapy for six years presenting with an acute intestinal obstruction, bowel hernia, bowel ischemia, and hemodynamic instability. CT abdomen revealed thickening of walls of colon and ileum in the right lower quadrant, with signs of small bowel obstruction. Patient underwent emergency laparotomy for the repair of hernia and resection of ischemic bowel, and intraoperatively, was found to have dusky, edematous, thickened, inflamed, and distended distal bowels with adhesions in the right lower quadrant. The pathological examination revealed a thin membrane encasing the ileum, colon and the mesenteric tissue diffusely. Microscopic examination of resected bowel showed marked submucosal edema with myxoid and inflammatory changes. Based on these clinical, radiological and pathological findings, a diagnosis of EPS was established. Her postoperative course was complicated by recurrent intraabdominal bleeding with hemoperitoneum, leading to disseminated intravascular coagulation, multiorgan failure, and death, two weeks after the surgery. EPS can present as an acute abdominal catastrophe. Although there are recommendations for ascertainment of EPS diagnosis, there are no clear guidelines for safe and effective surgical strategies and these warrant further research.

## Introduction

Peritoneal dialysis (PD) procedure is employed for the management of end-stage renal disease (ESRD) [1]. PD has been shown to improve the quality of life for ESRD patients; however, the procedure is associated with a distinct risk for abdominal complications [2]. While peritonitis is a common complication [3], encapsulating peritoneal sclerosis (EPS) is a unique, rare, but potentially fatal complication related to PD therapy [4]. EPS is progressive and is associated with high morbidity and high mortality, with 50% survival reported at one year after diagnosis [5]. According to the International Society for Peritoneal Dialysis (ISPD), EPS is “a syndrome in which adhesions of a diffusely thickened peritoneum causes repetitive and intermittent intestinal obstruction” [6]. The syndrome commonly presents insidiously with partial intestinal obstruction and malnutrition, but there is an intermittent, stepwise worsening with the advancement of the pathological process [7]. We report a case of EPS presenting as an acute abdominal catastrophe with intestinal obstruction, herniation, and ischemia leading to an emergency laparotomy, hernia repair, bowel resection, and postoperative bleeding, and death.

## Case presentation

A 60-year-old white female with ESRD on PD therapy for six years presented to our emergency room with fever, abdominal pain, drowsiness, nausea, vomiting, and diarrhea. She had a past medical history of tuberous sclerosis and renal angiomyolipoma. Additionally, she had childhood-onset seizure disorder related to tuberous sclerosis, associated developmental delays, and communication challenges. She required life-long special skilled nursing home care. There was no prior reported history of peritonitis. Physical examination revealed a temperature of 101.1 F, a respiration rate of 16/minute, a heart rate of 120/minute, and a low systolic blood pressure (BP) of 80 mm Hg with mean BP above 60mm of Hg, marginally low for her chronic state. On physical examination, her abdomen was distended and tender on palpation, and the bowel sounds were absent. The remaining examination was mostly unremarkable. Her blood investigation revealed hemoglobin of 10.1 g/dL, normal white blood cell count, and platelet cell count, potassium 3.1 mmol/L, bicarbonate 26 mmol/L, anion gap of 16, blood urea nitrogen level 77 mg/dL, serum creatinine 4.95 mg/dL, and a marginally elevated level of serum lactic acid (2.8 mmol/L (normal: ≤ 2 mmol/L). Although infective etiology was our first differential diagnosis, there were no obvious clinical signs to support a source of infection. The PD catheter exit site was unremarkable. The PD effluent showed slightly hazy fluid with microscopy showing evidence of only mild leukocytosis without neutrophilic dominance after two hours of dwell, and there was no hemoperitoneum. The PD fluid cell count and culture did not fulfill the diagnostic criteria for infective peritonitis. A slight elevation of white blood cell count raised concerns for sympathetic peritonitis (Table 1).

**Table 1:** Peritoneal dialysis fluid on admission and at 48 hours after admission.

Body fluid analysis	On admission	At 48 hours
Appearance	Hazy	Hazy
White blood cell count/µl	82	62
Polymorphonuclear cells (%)	4	3
Lymphocytes (%)	9	2
Monocytes (%)	89	93
Red blood cell count/µl	31	4

**Figure 1:**
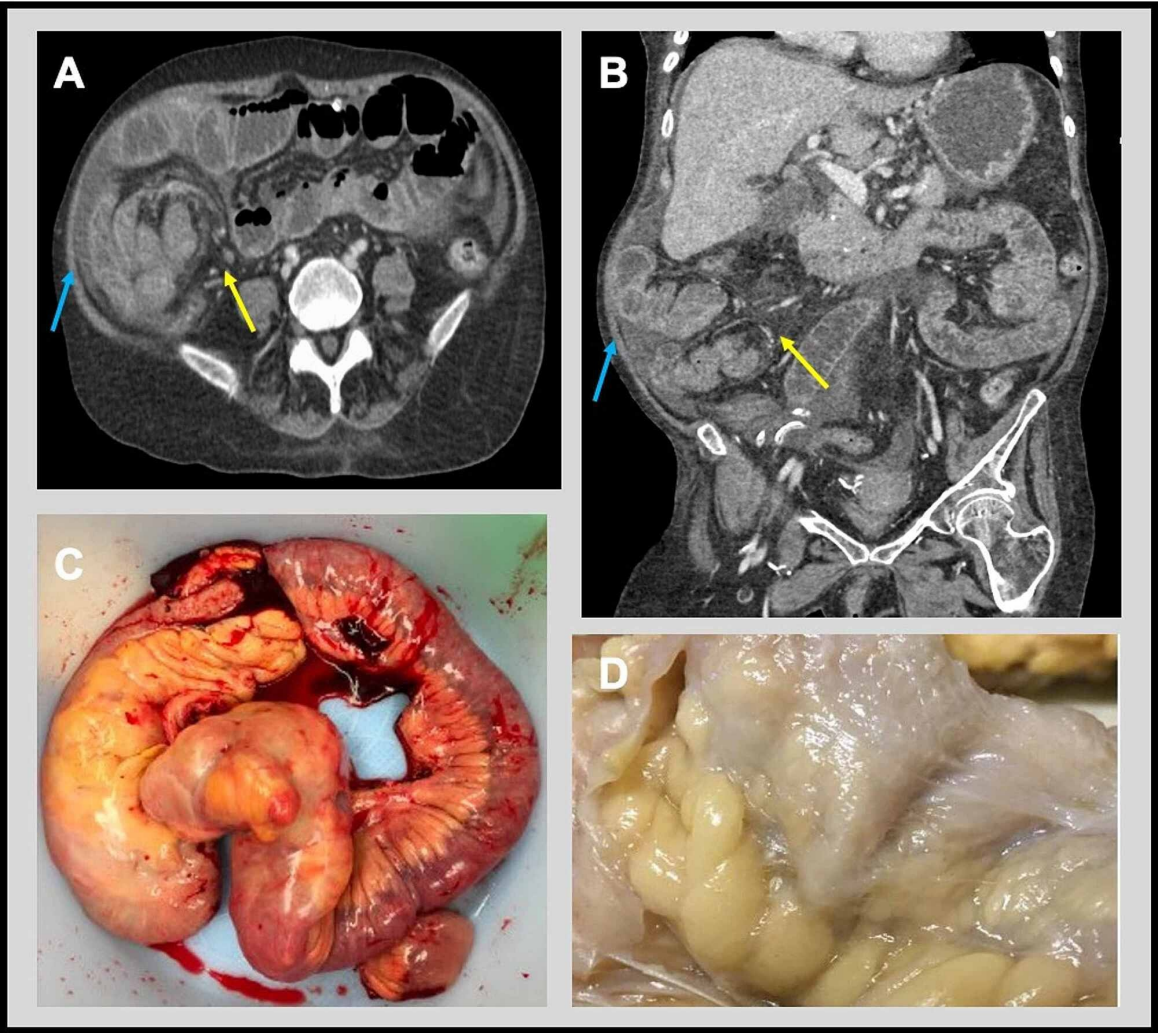
CT and gross pathology findings. CT abdomen/pelvis with IV contrast revealed thickening around the colon, dilatation of small bowel loops possible secondary to small bowel obstruction (blue arrow) with free intraperitoneal air and a transition point in the right lower quadrant (yellow arrow) with findings concerning an internal hernia, as well as pneumatosis in the cecum (A & B). Resection of ileum and right colon with serosa appearing rough in texture with light brown appearance characteristic in EPS (C). The ileum and colon segment has an extensively adhered focus of multiple loops of ileum just proximal to the ileocecal valve (23.0 cm from the proximal margin of resection and 25.0 cm from the distal margin of resection). The adhesion consists a semi-translucent membrane encapsulating the small bowel and its mesentery (D).

The blood and stool cultures were unremarkable. A chest X-ray ruled out an acute cardiopulmonary process. A CT scan of the abdomen revealed thickening around the colon and ileum in the right lower quadrant with signs of small bowel obstruction, hernia, and pneumatosis in the cecal wall (Figure 1A & B).

**Figure 2:**
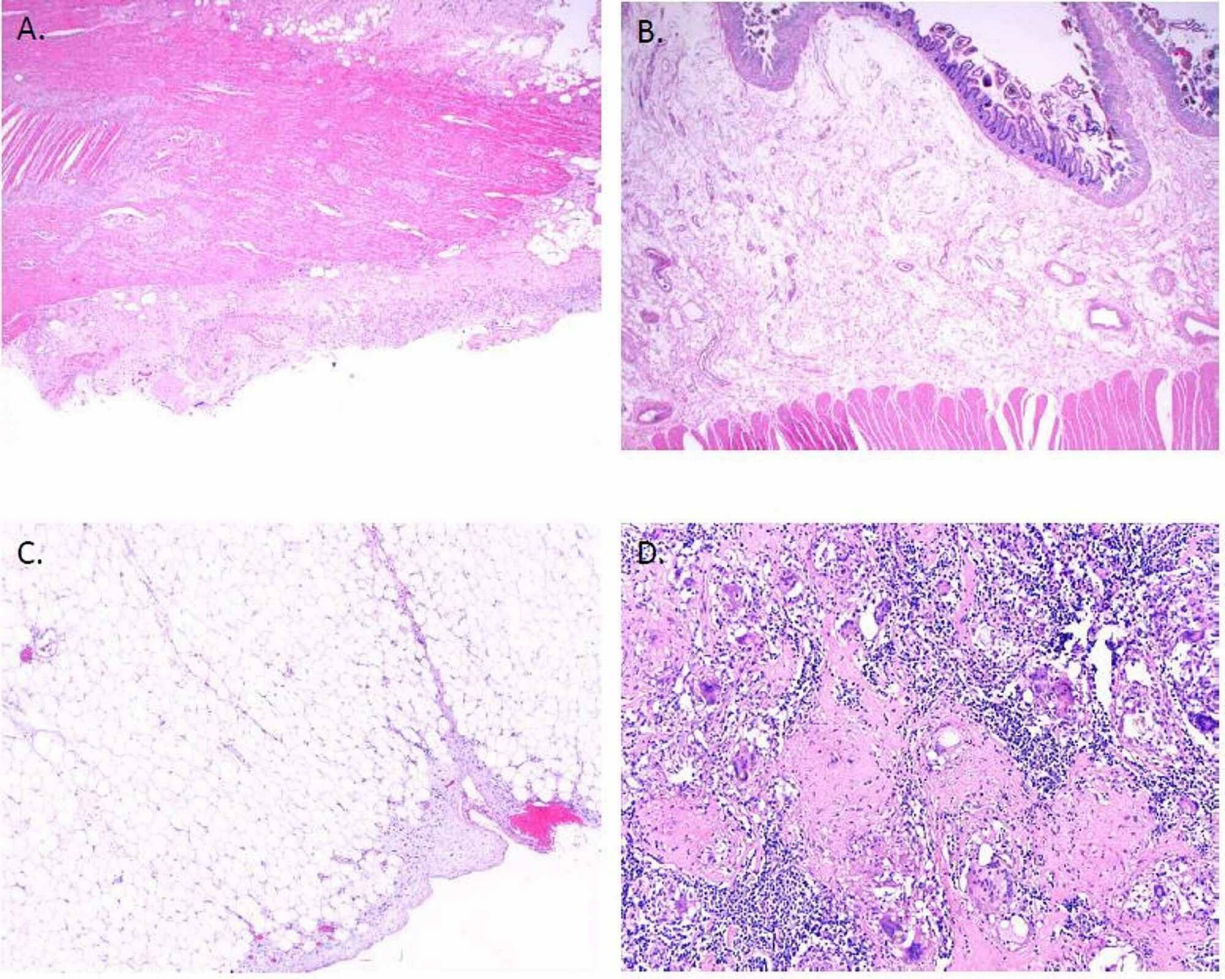
Histological findings of the resected bowel section. Histological examination of the bowel sections reveals delicate fibroblastic proliferation primarily underneath mesothelial lining (A). There is marked submucosal edema with myxoid changes and mild mucosal ischemic injury and old hemorrhage in the terminal ileum (B). There is mild mesenteric sclerosis (C). Multiple mesenteric lymph nodes show noncaseating giant cell granulomatous response to bifringent material and focal calcification (D).

She was started empirically on intravenous vancomycin and cefepime therapy. Despite 72-hour of antibiotic therapy in conjunction with nasogastric suction and hydration, she continued to remain febrile, tachycardic, and hypotensive. A repeat blood examination revealed persistent elevation of lactic acid levels (2.6 mmol/L). Due to concerns for internal bowel herniation and ischemia, she underwent an emergency laparotomy that revealed dusky, edematous, thickened, inflamed, and distended distal bowels with adhesions in the right lower quadrant. The mesentery attached to the bowels was also edematous, inflamed, and friable. She underwent resection of the ileum and right colon and removal of the PD catheter (Figure 1C). The immediate post-operative period was complicated by hemoperitoneum and gastrointestinal bleeding, requiring second emergency surgery. Despite these interventions, she continued to deteriorate. She developed hemodynamic instability and multiorgan failure and died two weeks later. Pathology of the resected specimen on gross examination revealed a relatively thin membrane encasing the ileum and colon, and the mesenteric tissue diffusely (Figure 1D). In the microscopic examination, the ileum and colon revealed marked submucosal edema with myxoid and inflammatory changes(Figure 2).

Together, her clinical, radiological, and pathological findings were consistent with a diagnosis of EPS.

## Discussion

EPS is a rare but serious manifestation of long-term PD therapy. The incidence of EPS increases with the duration on dialysis, and the risk of occurrence varying between 0.6% and 6.6% after five years on PD [5]. Some investigators have suggested younger age, use of conventional PD solution compared to biocompatible PD solution, prior history of abdominal surgery, recurrent or prior history of severe peritonitis, exposure to higher dialysate glucose, history of kidney transplantation, and ultrafiltration failure as additional risk factors [5].

Clinically, EPS presents insidiously, largely with abdominal symptoms of nausea, vomiting, and bloating, with or without small bowel obstruction, and symptoms related to chronic malnutrition [7]. There are no specific tests to diagnose EPS. Instead, a combination of clinical presentation and CT findings characterized by thickened enveloping peritoneum, and tethering of the small intestinal loops with accompanying bowel dilatation are employed to ascertain the diagnosis. Some experts have described a ‘peritoneal cocoon sign’ where the peritoneum wraps around the bowel as a hallmark feature for EPS though, this is not mandatory [8]. The PD fluid, when examined, commonly shows a varying degree of hemoperitoneum [9]. Pathologically, EPS involves a process of encasement of the intestinal loops with a fibro-collagenous membrane. In most cases, as the EPS progresses, PD is discontinued and the patient is transitioned to hemodialysis. However, the risk of developing EPS continues nearly five years after the PD therapy has been stopped. Management additionally requires nutritional support, sometimes parental nutrition. Some investigators have pursued steroids, tamoxifen, and other immunosuppressants; however, their overall utility is uncertain [10].

According to some investigators, EPS progresses through inflammatory, encapsulating fibrotic, and ileal stages of the disease, explaining the stepwise worsening of symptoms [10]. In some patients, the encapsulating stage may not be clinically significant if the capsule is too thin to impair intestinal peristalsis [5]. While the developmental limitations impaired the ability to communicate early symptoms, our patient had no known prior abdominal symptoms or peritonitis, and presented with an atypically rapid presentation. She was persistently febrile with elevated lactic acid and hypotension, but the labs and radiology did not support an infective etiology. The laparotomy procedure revealed dusky bowels and intestinal adhesions that explained the intestinal obstruction. Despite an emergency correction of hernia and resection of ischemic bowel, she developed hemoperitoneum.

Another important unmet need is guidance on the choice of surgical intervention for acute treatment of EPS [11]. Although our patient required repair of hernia and resection of ischemic bowel, a blunt dissection strategy or a laparoscopic procedure to perform lysis of adhesions could have minimized post-operative bleeding risk. Some specialized centers advocate for peritonectomy and enterolysis, a specific surgical choice accompanied by lower mortality and acceptable morbidity [12]. While ISPD has provided recommendations on prevention strategies for the development of EPS, there are no clear guidelines on a safe and effective surgical approach, and this warrants further research.

## Conclusions

EPS is a rare complication of PD therapy that can masquerade as an acute abdominal catastrophe. EPS involves an inflammatory and peritoneal fibrosis process that encases the intestinal loops with a thickened membrane. As the EPS progresses through various pathological stages, PD patients manifest clinical symptoms on an intermittent basis. In patients with the rapidly progressive pathological process, the clinical presentation can be acute and severe. A combination of abdominal symptoms and radiological features, along with a high index of suspicion, can establish a timely diagnosis of EPS. However, further research is needed to identify optimal and safe surgical interventions.
